# Health care professionals’ perspectives on self-management for people with Parkinson’s: qualitative findings from a UK study

**DOI:** 10.1186/s12877-021-02678-w

**Published:** 2021-12-15

**Authors:** Megan Armstrong, Remco Tuijt, Joy Read, Jennifer Pigott, Nathan Davies, Jill Manthorpe, Rachael Frost, Anette Schrag, Kate Walters

**Affiliations:** 1 Department of Primary Care and Population Health, University College London, Royal Free Campus, Rowland Hill Street, London, NW3 2PF UK; 2 Institute of Neurology, University College London, London, UK; 3 NIHR Health & Social Care Workforce Research Unit, King’s College London, London, UK

**Keywords:** Parkinson’s, Self-management, Qualitative, Thematic analysis

## Abstract

**Background:**

Parkinson’s disease is a long-term, complex health condition. To improve or maintain quality of life, people with Parkinson’s can have an active involvement in their care through self-management techniques. Given the complexity and individualization of self-management, people with Parkinson’s will need support and encouragement from their healthcare professionals (HCPs). Despite the key role HCPs have in this, research has seldom explored their perspectives and understanding of self-management for people with Parkinson’s.

**Methods:**

Multi-disciplinary teams providing care for people with Parkinson’s across London, Coventry and Hertfordshire were approached and took part in either one of four focus groups or individual interviews. Forty-two HCPs, including a range of specialist doctors, general practitioners, allied health professionals, nurses, and social workers, took part in this study. Interviews were transcribed and analysed using thematic analysis to identify themes.

**Results:**

Four themes were developed from the data: 1) Empowerment of patients through holistic care and being person-centred; 2) Maximising motivation and capability for patients, for example using asset based approaches and increasing opportunities; 3) importance of empowerment of carers to support self-management and 4) contextual barriers to self-management such as the social context.

**Conclusions:**

This study is the first to explore the perspectives of HCPs on self-management in people with Parkinson’s. Our findings have identified important considerations surrounding empowerment, motivation, carers and contextual barriers to better understand how we enable effective self-management techniques in people with Parkinson’s. Research should build on these findings on to develop acceptable and effective self-management tools for use in practice with people affected by Parkinson’s.

Parkinson’s is a common neurodegenerative disease affecting 1–2% of the population over the age of 65 years and, with increasing life expectancy, numbers are projected to double by 2050 [1]. It affects the individual in a myriad of ways, including movement disorders, cognitive decline and poor psychological wellbeing [2, 3]. Due to the frequency and severity of symptoms, people with Parkinson’s often have a low quality of life, increased risk of unplanned hospital admissions, and may move to care homes [4, 5]. It is important to understand how people with Parkinson’s can be best supported to self-manage their condition to maintain independence and optimise quality of life.

Self-management is the process of people with long-term health conditions actively participating in their care through adapting their behaviour to better manage their physical and emotional wellbeing [6, 7]. Specific aspects of self-management vary according to the condition, but many include access to health information and resources, monitoring of symptoms, problem solving and goal setting techniques, increasing self-efficacy and developing coping strategies [8, 9]. To begin self-management, people need knowledge, confidence, and skills; to self-manage long-term and adopt healthy behaviours, the person must focus on what intrinsically motivates them, which makes self-management individualized and therefore complex. Self-management has been shown to improve cognitive function in people living with dementia and enables them to live at home for longer [10], improve quality of life in people with multiple sclerosis by reducing depression and anxiety [11], and reduce pain in people with cancer [12]. Within Parkinson’s, evidence of the effectiveness of varieties of self-management interventions is limited [13]. Self-management techniques are often not implemented in practice and reasons as to why this is needs to be explored [14].

Complex trials are often not delivered in the ways participants want them to be [15] and the UK Medical Research Council’s guidelines highlight that a lack of effectiveness may be due to poor implementation and acceptability of the intervention [16]. Therefore, it is important to explore the perspectives of people receiving an intervention and other key stakeholders. A thematic synthesis of interviews with people with Parkinson’s and their family carers outlined several key components of self-management interventions such as increasing knowledge, developing psychological strategies, and providing self-monitoring techniques [17]. Whilst researchers have explored people with Parkinson’s and their carers’ perspectives of self-management, the role of those healthcare professionals (HCPs) who work in partnership with patients as a key component of self-management has not been greatly researched [18].

Relationships with HCPs is a major factor for effective self-management for people with complex long-term conditions [19]. This finding is unsurprising given that people are new to their role as active participants in care and may need guidance and support from HCPs. In a critique of self-management and behaviour change interventions, it was argued there is too much focus on the person with the condition changing their behaviour, and that HCPs are equally important in self-management [20]. One study that explored HCPs’ views of being person-centred, for people with Parkinson’s or those with diabetes, highlighted the barriers and uncertainty about knowing what was important to the patient [21]. More broadly looking at all aspects of self-management, HCPs for people with chronic obstructive pulmonary disease reported a lack of understanding of what it entailed and those who did understand it, felt they lacked the time to successfully implement it [22]. To our knowledge, there is no exploration of HCPs’ understanding of self-management specifically for people with Parkinson’s or their perspectives on the barriers and facilitators of implementing it in practice. Due to the increased interest in developing self-management interventions for people with Parkinson’s, it is important to understand HCPs’ views on self-management. We therefore aimed to explore the perspectives of HCPs on how to support people with Parkinson’s to self-manage their long-term condition.

## Method

### Design

An exploratory qualitative design was used with reporting guided by the Standards for Reporting Qualitative Research framework [23].

### Ethics and consent

This study was given a favourable opinion by the London Queen Square Research Ethics Committee (18/LO/1470). Written informed consent was obtained from all participants.

### Sampling and participants

A purposive sample of HCPs was approached and recruited in 2019 as part of the PD-Care study, which aims to develop a self-management toolkit for people with Parkinson’s. HCPs were selected to represent a broad range of community and hospital-based health and social care professionals who are involved in supporting people with Parkinson’s in one or more aspect of their health or social care. Four multi-disciplinary teams (MDTs) providing care for people with Parkinson’s across London and Hertfordshire were approached and agreed to host focus groups, including one General Practice team, a Community MDT, a hospital outpatient MDT and a hospital-based neurology team. To sample for maximum diversity of views based on professional background and level of experience a further 15 individual interviews were conducted with professionals under-represented in the focus groups. These individuals were recruited from a variety of hospital and community settings across London, Hertfordshire, and Coventry.

### Procedure

Interviews and focus groups were mainly conducted at the participant’s place of work. Interviews were semi-structured and followed a topic guide that included questions such as ‘How do you facilitate the self-management of patients with Parkinson’s disease?’ Further prompts and probes were used to facilitate in-depth responses. The study coordinator (JR), who has a background in nursing and psychology, and a research assistant who has a background in psychology, conducted the one-to-one interviews and focus groups. Both interviewers had experience in conducting qualitative interviews, which gave them the skills to build rapport with the participants to encourage open and honest answers. Techniques such as paraphrasing and summarising throughout was used to allow the participants to correct the interviewers or elaborate on their answers. Moderation of the focus groups was agreed upon concerning a ‘step-up, step-down’ approach to give everyone a chance to participate. The interviews lasted between 60-90 minutes. Discussions were audio-recorded, transcribed verbatim, de-identified and stored securely. All transcripts were checked against initial recordings before processing for analysis.

### Analysis

Thematic analysis guided by Braun and Clark’s framework [24] was used to identify, analyse, and report themes. Line-by-line coding was undertaken using NVIVO 12 [25]. An inductive approach was taken, where different members of the team read several transcripts multiple times to identify codes from the data. Codes were then synthesised into the themes and subthemes and discussed between the whole team, including five HCPs, to explore and agree meanings and interpretation.

## Results

### Participant characteristics

This study included 42 HCPs, including a range of specialist doctors, general practitioners (GPs), allied health professionals, nurses, and social workers (see Table 1).

**Table 1:** Participants’ professions

ID	Profession
Focus group 1: GP practice	Practice nurses x 2 Health Care Assistant x 1 GP x 6 GP Trainees x 2
Focus group 2: community based outpatient MDT	OT Consultant team lead x 1 Physiotherapists of differing grades x 3 Therapy assistant x 1 Speech and Language Therapist x 1 Continuing care nurse x 1 Community Matron x 1
Focus group 3: hospital clinic based MDT	Parkinson’s disease nurse x 1 Clinic nurse x 1 Consultant neurologist x 1 Physiotherapist x 1 Consultant Geriatrician x 1 Occupational Therapist x 1
Focus group 4: Hospital based neurology team	Neurologists x 2 Parkinson’s Disease Nurse x 1
Individual interviews (*n*=15)	Community speech and Language Therapist x 1 GP x 1 Trainee Neurologist x 1 Neurologist consultant (non PD specialist) x 1 Foundation Doctor (junior trainee) x 1 Hospital pharmacist x 1 Community Occupational Therapist/Care Co-ordinator x 1 Trainee geriatrician x 1 Hospital Social Worker x 1 Community Social Worker x 2 Community dietician x 1 Hospice based Palliative care nurse x 1 Clinical psychologist x 1 Consultant Psychiatrist (older adults) x 1

### Findings

Four main themes were identified that centred on empowerment of people with Parkinson’s, increasing motivation and capability to self-manage, the family carers’ role in self-management, and contextual barriers to self-management (see Table 2).

**Table 2:** Themes and sub-themes

Themes	Sub-themes
**Empowerment**	Holistic care Person-centred
**Increasing motivation and capability to self-manage**	Maximising motivation Asset-based approaches Maximizing capability and opportunities
**Carers’ role in self-management**	Self-management with carers Empowering carers
**Contextual barriers to self-management**	Social context HCPs’ skills and knowledge to support self-management

#### Empowerment

HCPs discussed ways in which they were already supporting people with Parkinson’s to self-manage their condition, which centred on what they described as ‘empowering’ individuals. Key components of empowering people with Parkinson’s to self-manage were holistic and person-centred care.

##### Holistic care

Providing holistic care (i.e., care that incorporates the physical, psychological, and emotional symptoms and therefore treating the person as a whole) and developing holistic self-management plans were considered important by most participants:

*“I think I got the most useful feedback when people with Parkinson’s disease had been approached in a holistic way I think. When they say you know it wasn’t just somebody looking at my memory, or somebody looking at this, my cognition. It was seeing me as a person and not as you know a diagnosis, or pathologising what I’m going through. So, I think the feedback I’ve had from people is don’t just look at one thing, look at everything. Look at me as a human being, which is strange to say, but you know I don’t think people feel like that in the NHS sometimes. We always have services separating everything.”* Clinical psychologist

Some HCP highlighted the importance of people with Parkinson’s taking a holistic view themselves too:

*“It is about the self-management. It is about looking after yourself emotionally and psychologically, as well as physically. So, good mental health, good social activity, good physical activity. Looking at dietary intake, fluid intake. Not ignoring the fact that you have other health conditions and not blaming everything on the Parkinson's, I think is a big thing.”* Focus group - hospital based outpatient MDT

##### Person-centred

To support people to self-manage their Parkinson’s, around half of the HCPs highlighted the importance of being person-centred. Below a HCP described how being person-centred means focusing on what is important to the person and differentiated between what they considered to be the medical and social model:

*“….so many people are so used to the medical model and being asked questions and answering that question and stopping. I think it’s worthwhile having quite an open space and saying you tell me what’s important, and you tell me, you know, what are your obstacles, and it can be anything.”* Clinical Psychologist

Self-management was conceptualised by some HCPs as working with patients as partners in their care to offer them choices:

*“We don’t do things for people. We don’t impose. We work together as partners. So, if I’m setting somebody’s care plan, putting somebody’s care plan in place, I will ask them, what would you like?”* Social worker 1

One HCP highlighted the importance of why patients should be empowered to self-manage their condition, particularly as there is insufficient time for many HCP to provide ongoing management guidance, and noted that many already were doing this:

*“As far as I’m concerned, I see them for 15 minutes twice a year, and they then spend however many thousand hours a year without me to guide them or to tell them what to do. So they are much more likely to have figured out what works for them than I am in those 15 minute slots, so I’m a big advocate of them working out what works for them.”* GP

Being person-centred did not mean setting unrealistic goals just because they are considered a priority by the person with Parkinson’s, and again links to the idea of a partnership approach between the HCP and the patient:

*“Instead of asking somebody how they live their best life, I would say to them at this moment in time, what are you doing? What is your typical day like at the minute? We used to say, in an ideal world, what you like to be doing? But again, people would be like, oh, I’d like to be on a cruise in the Caribbean. Quite clearly, wouldn’t we all like to do that?”* Social worker 2

Self-management materials that may be developed were considered to be person-centred if they were tailored to individuals. However, while information should be evidence-based it had to be individually adapted:

*“It should be evidence-based….so we all go through what has been previously proven. Different things work for different people…it’s just then picking out things that work for some people, but not for others.”* Speech and Language Therapist

#### Increasing motivation, and capability to self-manage

##### Maximising motivation

A common concern among HCPs was that some people seem to lack motivation to self-manage their condition.

*“She was quite inactive about self-managing her condition. Yes, she was not motivated at all… Sometimes they haven’t got anybody else to sort of motivate them as well….And we have people where almost every session or every episode of care, they will say, “I know nothing about Parkinson’s.” And you think, “I’m sure you do. It has been explained.” But, you know, they’re not ready or not engaged….But I mean, I don’t think… perhaps it could be any diagnosis and it would be similar, you know.”* Focus group: hospital based outpatient MDT

In addition, participants in another focus group drew attention to decreased motivation as part of the Parkinson’s symptoms, which highlights a need for personalized self-management tools to incorporate such changes:

*“The, the other kind of thing is obviously that motivation is a huge factor for PD anyway, because the condition itself. And I, I sometimes feel that you’d be pushing the goal, not the patient. So it’s, it’s not particularly personalised I guess, to a certain extent.”* Community MDT

Motivational interviewing was mentioned as being a useful technique to encourage people with Parkinson’s to self-manage, although little detail was given about *how* professionals were using motivational interviewing. Most HCPs did not define motivational interviewing but the HCP below describes using it to identify internal motivations for the patient:

*“Motivational interviewing with people who have a progressive condition is very challenging. But you can always find something that they can latch on to….It’s just thinking about having a really good social history and trying to think, what is it that gets them up in the day, whether it’s they have a hobby they do with their spouse, or whether they enjoy spending time with their grandchildren, gardening, whatever it is, trying to not use it, but empower them to think that if they’re nutritionally well, they’d be in a better place to do those things.”* Community Dietician

Another technique used to encourage motivation when it seemed lacking in the patient was to take it slowly and encourage the patient to gradually get involved in a task until they were able to achieve the task independently:

*“The hospital nurses, even before the specialist nurses come in, they can have that conversation with the patient, “this is what I’m doing, this is how you do it,” rather than just doing it without explaining it. Especially if the patient wants to be involved….I had a patient who was very reluctant initially, but then as time progressed, because he’s like, I’m not a nurse, I’m not getting involved, I’m not going to be able to do it, he has this whole anxiety about it. As time progressed, he got involved little by little, by the end he was doing it completely independently. It was quite a change, it was really good to see.”* Community Dietician

For others, a more explicit consideration of the psychology of motivation was important, and professionals could help the motivation to change become intrinsic:

*“Because I think sometimes with behaviour the change has to come from within, and with really coming to a place of acceptance as well about that, in order to use something. To use strategies and things like that, and to be able to want to take on information.”* Clinical psychologist

In order to be intrinsically motivated to change it was noted by some that a person with Parkinson’s needed to have a sense of self-efficacy; that is a belief in their own psychological capability to change:

*“So I think an element of it is…whether they feel they have an ability to change things or not with their own actions. It varies between people.”* Trainee geriatrician

##### Asset-based approaches

HCPs individually varied in whether they preferred to focus on patient assets or strengths (i.e., what they can do) or their deficits (i.e., what they cannot do) to elicit self-management. In contrast to standard medical care, which tends to be led by a deficit-based model and solving problems, most HCPs, thought promoting assets in self-management was more effective at increasing motivation:

**“** *I don’t like to use negative approaches, I think “we want to prevent you from having a fall” would be a complete fail. I’m thinking, if they do have hobbies, just being able to go and do their hobbies.”* Community Dietician

However, a few HCPs felt emphasising consequences (what could happen if they did not engage in a behaviour) could be good motivators:

*“We use motivational interviewing…and say, okay, you were able to do this and that, so what’s stopping now from doing that, and what do you think should be done? Continue showering. Continue washing yourself because you lose your dignity if somebody else come and wash you. Would you like that? Would you like somebody to come in and wash you?”* Social worker 3

##### Maximising capability and opportunity

It was noted that to achieve any task, people with Parkinson’s need to be knowledgeable about what to do, how to do and why it’s important:

*“Therefore that is the importance of self-managing your condition and taking some ownership over it in terms of them making sure if you want to get up at 7.30 in the morning, well maybe you can self-administer and give it (medication) to yourself at 7.30 in the morning. But to do that, to enable you to do that, you need to know about your medicines and they might not know about it.”* Pharmacist

However, others felt that providing too much information at once that often happened in practice could be overwhelming and was therefore an ineffective way to support self-management:

*“…I think we do bombard people with a lot of information. And, you know, that’s when you come back a couple of weeks later and it almost looks like, they just don’t remember very much…they’ve just had a lot of information from the consultant, a lot of information about their medication, a lot of information about the diagnosis. And then we just come with more and more and it must be really overwhelming.”* Focus group: hospital-based outpatient MDT

Capability to self-manage in late-stage Parkinson’s was highlighted as a particular challenge although not discussed in depth.

*“I think it’s very difficult. I have to say. I don’t, I don’t know whether, um, they do effectively self-manage in the end. I don’t know.”* Community MDT

Other HCPs highlighted ways to support and encourage motivation in people with complex or late-stage Parkinson’s:

*“And it’s about empowering the person, even if they do have confusion or if they are struggling. I can go and see somebody who can't always have a conversation, but I will still sit and have half an hour, even if they don’t understand what I'm saying, I’ll still ask them questions and see how they respond and things.”* Social worker 2

It was also noted that for someone whose advanced symptoms might have reduced intrinsic motivation, perhaps due to apathy related to their condition, extrinsic motivators and reminders become more important:

*“And if you’re feeling apathetic, then you’re not going to think, I am feeling apathetic. I must go and get my Parkinson’s book and find out why I’m feeling apathetic, and that will tell me what to do. Because you’re not going to….So that needs to come from the outside. Now, that could be something as minimal as an alert coming up on your phone saying, ‘it’s June 16 today’s the day that you should look at page 12 of your self-management guide’.”* Trainee Neurologist

Some HCPs recognized that people with Parkinson’s might face barriers to doing certain activities, which might appear as a lack of motivation but were due to other factors such as lack of opportunity.

*“But motivation sometimes is not, I don’t know, because motivation sometimes would just imply that the person is not motivated, but sometimes, I think even for me, as a therapist, it’s really hard to, to find the motivation. You know, we have a lot of people who live by themselves…The best speech therapy is to speak. And you have nobody to talk to. So what therapy are we doing here?”* Community MDT

#### Carers’ role in self-management

##### Self-management with carers

HCPs frequently discussed carers and their role in self-management:

*“Because if it is self-management it doesn’t just have to be specifically for the patient….The things that could either be, sort of carer specific, um, in terms of when you’re with your relative or the person with Parkinson’s, you start noticing things like this have come along, you might be more at risk of a fall. And then the guidance or advice for the next sort of stage.”* Community MDT

Indeed, some HCPs based at a hospital felt that it would be helpful to give any information or guidance to the carer instead of the patient with a view that it is the carer who generally manages physical care rather than the person with Parkinson’s:

*“it’s the family who usually maybe monitor their medication, monitor their bowels or anything….It’s not the patient actually who is going to read about it, but the family member who is supporting the patient.”* Focus group: hospital based outpatients MDT

Some were aware of the risk that self-management could possibly exacerbate carers’ work. One HCP and displayed concerns about self-management:

*“I guess they’ve got to have adequate support as well otherwise the burden just gets shifted on to them [carers] rather than… Sometimes I think this self-management is a way of kind of just shifting the burden.”* Consultant psychiatrist

As highlighted above, HCP often discussed motivating patients and here HCPs highlight the role carers have in that. Some HCPs felt if it were not for carers, tasks would not get done due to the task not being prioritised by the patient:

*“…because the carers are often children … sometimes, it would be the children motivating them to do that sort of thing. Because otherwise, I don’t think they would have the motivation or whatever.”* Occupational Therapist

It was recognised that those patients without family carers would need more support or monitoring when self-managing and so extra support or monitoring was thought necessary for people without supportive social networks:

*“So I think that's probably where the emphasis should fall more on us as healthcare professionals and social workers as well to make sure that we're doing probably a bit more to help them….Whether it's simple things, like if their motor symptoms are getting to a stage where actually they can't really cook for themselves, then we need to make sure that they are eating and drinking and getting all the nutrition that they require.”* Foundation doctor

##### Empowering carers

Some HCPs felt that carers need to feel empowered by self-management resources. A trainee neurologist discussed why it is important to empower carers but did not provide specific techniques of *how* that should be done:

*“Carers of people with advanced Parkinson’s have got an awful life themselves, and how they can get support and how they should feel empowered to go and ask for support so that they can then continue to look after their partner better and more of the time as opposed to struggling all of the time.”* Trainee Neurologist

One HCP suggested that involving carers in the self-management of the patient and drawing upon their own assets as carers (i.e., what they do well already) can empower them:

*“And supporting their spousal carers to help them with that [self-management] as well because I think they can be feeling powerlessness and feeling useless in not being able to help. I think some of that was trying to empower people. There are things you can do. There are things you can help yourself, probably things you might be doing already that are evidence-based and important.”* Clinical Psychologist

Another suggested providing carers with more knowledge of how to provide the best care was another route to empowering them:

*“….a lot of the carers think they’re kind of miracle work and a fixer of all problems… but I always try and empower them and make them think, actually, if you do food first, you can achieve a lot more than just putting a person into (food supplement) 4 to 6 a day.”* Community Dietician

#### Contextual barriers to self-management

##### Social context

For self-management to be successful, it must work within the social context in which the patients live and access health care. Whilst it did not appear to lead to a sense of futility in promoting self-management, it did cause challenges. Here one HCP reflects on the broader aspects that need to improve to support people to self-manage:

*“Better treatments, disease modification, a change in social attitudes towards aging and infirmity, better support for people as they get some degree of cognitive impairment, better support for caregivers, I mean big, big societal stuff, the stuff that isn’t easy to treat, the kind of thing that makes disease modifying drugs look easy.”* Trainee neurologist

Another barrier to self-management can be the inflexibility of the health care system:

*“Because we talk about self-management and how do I encourage the patients to not put all their medications in all their little plastic pots with their own little label system. Because they come in with those, and legally they cannot be dispensed and administered. It’s very hard to really optimise their normal self-management within an institutionalised organisation.”* Focus group 4: Hospital based neurology team

Additionally, the health care system is fragmented and various services can work differently:

*“…the general divide which is the NHS and Social Services. It’s always been the same, and nobody chooses it to be there, but we work by the social model, they work with the medical model, so we work quite differently…Medical model is usually symptom-led and diagnosis-led really.”* Social worker 1

One HCP highlighted that the impact of defragmented services is a loss of information.

*“And I wonder if there could be some more joined-up working, and to know what that would look like….because it feels like a lot of information can slip through the net.”* Clinical psychologist

A lack of shared patient records meant HCPs did not always have access to the same materials or sources of information that the patient has to support them to self-manage:

*“I think it's also quite helpful to have…information on the patient themselves. So if there was somewhere that had who their clinician was, how long they've had Parkinson's and what their main symptoms are, what meds (medications) they're on, what meds they've previously had. So it's kind of a general overview. [..] That would be useful.”* Foundation year doctor

Furthermore, HCPs time with patients is very limited:

*“I think the real frustration, not for us, but for patients, you know, how many times more, you know, there’s this kind of a huge hiatus, because actually things change from day to day, you know, they literally change from day to day, minute to minute. But, you know, a 6-monthly appointment, which is probably all you are resourced to do, is meaningless actually for people in their day-to-day life.”* Focus Group 1: GP practice

##### HCPs’ skills and knowledge to support self-management

HCP acknowledged several limitations in their capability to support self-management. Some of those interviewed did not see many Parkinson’s patients in practice and, together with the complexity of the disease, this group found it challenging to support people with Parkinson’s to self-manage especially at later stages:

*“People, you know, again people, there aren’t maybe vast numbers, people are at different stages, some people who are housebound, some people are mobile, some people are working. So I just find it hard to know how it would work in practice really.”* GP

If there were no specialists available for advice or if someone had not had much or recent training on Parkinson’s, HCPs had to seek out their own information online:

*“So I haven’t had any Parkinson’s training since I’ve been here and I’m not sure I’ve had any ever. It’s kind of just that you do your own, like thinking that, oh, I haven’t looked after someone with that condition for a while, so kind of going back and doing a bit of your own research and your own reading.”* Palliative care nurse

Occasionally HCPs displayed a lack of understanding on how to motivate people to change and overcome barriers in capability:

*“But the patients also expect a lot, by the way. They become really lazy. So a lot of patients say, oh, we’re only given a leaflet….I say to them you’re full-time at home, so make use of your time.”* Neurologist consultant

## Discussion

To the best of our knowledge, this is the first study to explore HCPs’ perspectives of self-management in people with Parkinson’s and its implications in practice. We identified four main themes covering empowerment, increasing motivation, capability and opportunity, carers’ roles in self-management and contextual factors. Unlike evidence from people with Parkinson’s who discussed the core components of self-management such as providing information and developing skills [17], HCPs were more focused on the contextual barriers and how to enable self-management. Many HCPs highlighted the importance of being holistic and person-centred. By person-centred, HCPs interpreted this as working in partnership or led by the person with Parkinson’s and what is important to them whilst creating realistic and manageable goals. Around half the participants highlighted being person-centred and appeared to incorporate this into their practice. However, there was a minority who did not appear to be person-centred indicated by using terms such as ‘lazy’. To be person-centred, HCPs needed the time and a willingness to explore what ‘living well’ looked like for each person. In practice this can be challenging for some HCPs who get very little time with the patient.

Motivation, as acknowledged by some HCPs, can be low in people with Parkinson’s, and adds an additional challenge in self-management [26]. Low motivation can be due to the motivation not being intrinsic, a symptom of Parkinson’s, as well as a lack of capacity or opportunity. Motivational interviewing was mentioned several times to increase motivation, which was described as identifying intrinsic goals for the patients (i.e., doing something because it is enjoyable or important not for external rewards). Motivational interviewing incorporates other aspects such as empowerment and empathetic listening [27]; however, it was not possible from the data to interpret whether HCPs used these elements in their practice. Motivational interviewing is an important technique in promoting self-management and has shown to be beneficial for encouraging adherence to medication in people with a range of chronic conditions [28] and exercise programmes for people with Parkinson’s disease [29].

Although most HCPs stated they focused on tasks or goals that patients are able to do, some still focused on things patients cannot do or may not be able to do in the future; these approaches are known as asset-based and deficit-based, respectively [30]. Asset-based approaches to health promotion have been shown to be beneficial for patients, but more research is needed to explore their place in self-management and for people with Parkinson’s [31]. Beyond motivation, HCPs also mentioned maximising capability and opportunity by increasing patients’ knowledge gradually, ensuring they have self-efficacy and increasing external motivators or reminders in the later stages of the disease.

Whilst some HCPs acknowledged that self-management might compound the burden of work on carers, others discussed working with the carer instead of the patient in situations where it was felt the patient may not engage or understand. The reasons patients might not engage was not discussed; however, it is possible the HCPs were not being entirely person-centred in these scenarios. In these interviews, the same HCPs who discussed being person-centred with the patient, were also the ones to consider carer burden and wellbeing. Finding the balance between integrating carers in self-management but not increasing their burden is of high importance, as carers have expressed feeling excluded in the caring decisions and journey of the people they support [32]. HCPs suggested empowering carers through increasing their knowledge and focusing on their assets.

There was a suggestion from some HCPs that doctors, both in primary and hospital care, are focussed primarily on diagnosis and symptom management. Previous research has highlighted HCPs can be unsure about how to enable patients to self-manage [21]. Together with the limited time available with HCPs and the inflexible, stretched health care system, self-management may not have a defined role in medical care. It is possible there may be some HCPs better placed to support with self-management such as Parkinson’s disease nurse specialists who have the most contact with people with Parkinson’s and understanding of the condition. However, from our study, whilst we cannot draw concrete conclusions based on the breath of HCPs interviewed, it appeared the skills and willingness to engage in self-management was more person dependent than profession dependent. For example, one GP highlighted because their time was limited with a patient, they were then led by the person with Parkinson’s in identifying the problem and treatment.

Across all HCPs there was less overt reference to Behavioural Change Theory and problem solving than to motivational interviewing. If we are to expect HCPs to proactively support patients to self-manage, more training is needed about how to implement motivational, person-centred techniques. . Additionally, any self-management resources need to fit the patients’ and HCPs’ societal context such as the health and social care system supporting previous research that has highlighted the need for self-management interventions to work with HCPs and within the context also [20]

The strength of this study is the inclusion of a wide range of different professionals, providing a breath of opinion. We were able to interview HCPs from different localities, individually and in groups. Furthermore, the interviews were analysed by a range of researchers and HCPs providing an in-depth interpretation of the data. Our main limitation is that the findings here may not represent the views of HCPs from other areas and that interviews may reflect an idealised account of practice. Due to the variety of HCPs included in the study, we did not have the quantity to make comparisons between the professionals. Future research might explore how our findings differ by various HCPs. Additionally, most participants were female, although no obvious differences between genders were identified in our data but these and other socio-demographic factors could be explored with a wider sample.

This study is the first to explore the perspectives of HCPs on self-management in people with Parkinson’s, which, despite the key role HCPs have in self-management, has been largely neglected. Our findings have identified the impact of empowerment and motivation of people with Parkinson’s, the importance of the carer, and the contextual barriers there are to enabling self-management. HCPs were open to incorporating self-management in their clinical practice, despite the challenges. Research should build on these findings to develop acceptable and effective self-management tools that incorporate the perspectives of people with Parkinson’s as well as HCPs.

## Data Availability

The datasets analysed during the current study are available from the corresponding author on reasonable request, and subject to ethics and data protection requirements.
